# Assessment of diaphragmatic thickness by ultrasonography in Duchenne muscular dystrophy (DMD) patients

**DOI:** 10.1371/journal.pone.0200582

**Published:** 2018-07-26

**Authors:** Marianna Laviola, Rita Priori, Maria Grazia D’Angelo, Andrea Aliverti

**Affiliations:** 1 Department of Electronics, Information and Bioengineering, Politecnico di Milano, Milan, Italy; 2 Department of Neurorehabilitation, Scientific Institute IRCCS Eugenio Medea, Bosisio Parini, Italy; University of Minnesota Medical Center, UNITED STATES

## Abstract

**Introduction:**

In Duchenne muscular dystrophy (DMD) the assessment of diaphragmatic function is crucial because respiratory muscle weakness can cause respiratory failure. We aimed to noninvasively assess diaphragmatic function in DMD by measuring diaphragmatic thickness by ultrasonography, under the hypothesis that the progressive decrease of lung function is related to alterations of diaphragmatic thickness.

**Methods:**

Forty-four DMD patients and thirteen healthy controls were enrolled and subdivided into three age groups. Diaphragmatic thickness was measured during quiet breathing, inspiratory capacity, maximal inspiratory pressure and expiratory pressure maneuvers.

**Results:**

In DMD, absolute values of diaphragmatic thickness were significantly lower than in controls in the majority of the manoeuvers and diaphragmatic thickness significantly decreased with age at end-expiration, remaining constant at end-inspiration and during maximal inspiratory pressure maneuvers. Comparing to controls, absolute values of diaphragmatic thickness and diaphragmatic thickness variations were significantly lower (p<0.001), with the exception of quiet breathing and maximal expiratory pressure maneuvers in the youngest DMD. During maximal inspiratory pressure maneuver, variation of diaphragmatic thickness was not significantly different in the all groups, nevertheless maximal inspiratory pressure decreases with age.

**Conclusions:**

The diaphragm is prone to pseudo-hypertrophy in the youngest DMD, and to progressive atrophy in middle-age and oldest DMD. Diaphragm impairment could be expressed as a dissociation between muscle drive and muscle developed force. Ultrasonography could be used as a noninvasive method to assess progressive diaphragmatic weakness.

## Introduction

Respiratory failure is the commonest cause of death in Duchenne muscular dystrophy (DMD) and it is caused by progressive respiratory muscle weakness, which tends to develop only at the pre-terminal stage of the illness [1]. The assessment of diaphragmatic function, as the main inspiratory muscle, thus results to be of extreme importance, but the techniques traditionally employed to assess diaphragmatic weakness or paralysis in DMD, such as transdiaphragmatic pressure, EMG, fluoroscopy and plethysmography are either highly invasive, associated with radiation or very complex.

A hallmark sign of DMD is the progressive atrophy of the skeletal muscles, together with the so-called ‘pseudo-hypertrophy’, which is caused by replacement or infiltration of muscles by fatty and/or collagenous tissue and is present in specific muscle compartments. This information regarding the structural alteration of the diaphragm comes from an autopsy study performed on *mdx* mice [2], due to the inherent difficulties in performing in-vivo studies in humans. Computed tomography (CT) and Magnetic Resonance Imaging (MRI) provide good resolution and allow to obtain a detailed 3-D reconstruction of the shape of the diaphragm [3–5]. However, the clinical use of volumetric techniques based on CT and MRI is still limited for several reasons, namely the ability to analyse only horizontal postures, the high costs, the radiation exposure in CT, and the prolonged timing required for data acquisition in MRI. Ultrasonography (US) has proven to be useful for the study of anatomical characteristics of many muscle groups [6–9] and has been proposed as a possible alternative to study both diaphragmatic structure and function, namely diaphragmatic thickness [9–11], thickening ratio in adults [12] and children [13] mechanically ventilated and excursion [14, 15].

The aim of this study is to noninvasively assess diaphragmatic function in DMD patients by measuring diaphragmatic thickness (DT) by US, under the hypothesis that the progressive decrease of lung function is related to alterations of DT.

## Materials and methods

### Subjects

A total of 57 subjects were enrolled for the study, 44 DMD patients and 13 age-matched healthy controls. DMD patients were selected according to the following inclusion criteria: free of non-DMD respiratory complications, older than 6 years and able to perform respiratory maneuvers. The diagnosis of DMD was made on the basis of traditional criteria, i.e. progressive muscular deficit resulting in severe motor disability, increased muscle plasma enzymes, muscle biopsy identifying muscular degeneration and absence of dystrophin, alterations in the DMD gene (deletions, duplications or point mutations).

DMD patients and controls were subdivided into three groups according to age: <14 yrs (4 still ambulant), between 14 and 18 yrs and >18 yrs old. This subdivision of our patients is related to disease stages according to published natural history data (< 14 yrs: loss of ambulation and initial respiratory function decline; 14–18 yrs, respiratory function alteration associated to global moderate-severe muscular involment; < 18 yrs advanced stage of the disease, need of ventilator or cough device support) [16, 17]. Control subjects were selected based on the following criteria: no history of smoking; no previous lung, orthopedic or rheumatologic disease or spinal deformities that compromised respiratory system mechanics; not having undergone a specific sporting training. All subjects (or parents of the patient in the case of children) signed a written informed consent form. The study was approved by the Ethical Committee of the IRCCS ‘‘E. Medea” Institute according to the declaration of Helsinki.

### Pulmonary function tests and respiratory muscle assessment

Forty-two DMD patients underwent pulmonary function tests. Measurements of forced vital capacity (FVC), forced expiratory volume in 1s (FEV1) and peak expiratory flow (PEF) were performed in a seated position with a flow meter attached to a flanged rubber mouthpiece, with the nose occluded (Vmax series 22; SensorMedics, Yorba Linda, CA, USA). Subdivisions of lung volumes (functional residual capacity (FRC), residual volume (RV) and total lung capacity (TLC)) were measured using the nitrogen washout technique (Vmax series 22; SensorMedics). Nocturnal oxygen saturation measurements by pulse oximetry (Nonin 8500; Nonin, Minneapolis, MN, USA) were also performed in all patients. Maximal respiratory pressures were measured at the mouth (MicroRPM; Micro Medical Ltd., Rochester, England) in seated position. Maximal expiratory pressure (MEP) and maximal inspiratory pressure (MIP) were performed starting respectively from TLC and RV and the effort was maintained for at least one second. The best MEP and MIP values in two or more attempts were chosen.

### Ultrasound assessment of diaphragmatic thickness

In all subjects, DT was measured in supine position by ultrasonography during 1 minute of quiet breathing (QB), 2 inspiratory capacity (IC) maneuvers (full inspiration from Functional Residual Capacity, FRC, to Total Lung Capacity, TLC), 2 maximal inspiratory pressure (MIP) maneuvers, performed at residual volume (RV) and 2 maximal expiratory pressure (MEP) maneuvers, performed at TLC. A standard echograph (Aquila Esaote, Genoa, Italy) equipped with an 8 MHz linear probe was used. The probe was placed on the lateral ribcage in the right 9^th^ or 10^th^ intercostal space between the midclavicular and anterior axillary lines and it was firmly held in this position during each maneuver. High resolution B-mode allowed to visualize the diaphragm, identified as the region between two clear bright parallel lines, namely the pleural and peritoneal membranes. Flow and pressure were measured at the mouth respectively by a pneumotacograph (3813, Hans Rudolph, Kansas City, Missouri), connected to a low range pressure sensor (RCEM010DB, Sensortechnics, Munich, Germany), and a high range pressure transducer (RCEM250DB, Sensortechnics, Munich, Germany).

**Fig 1:**
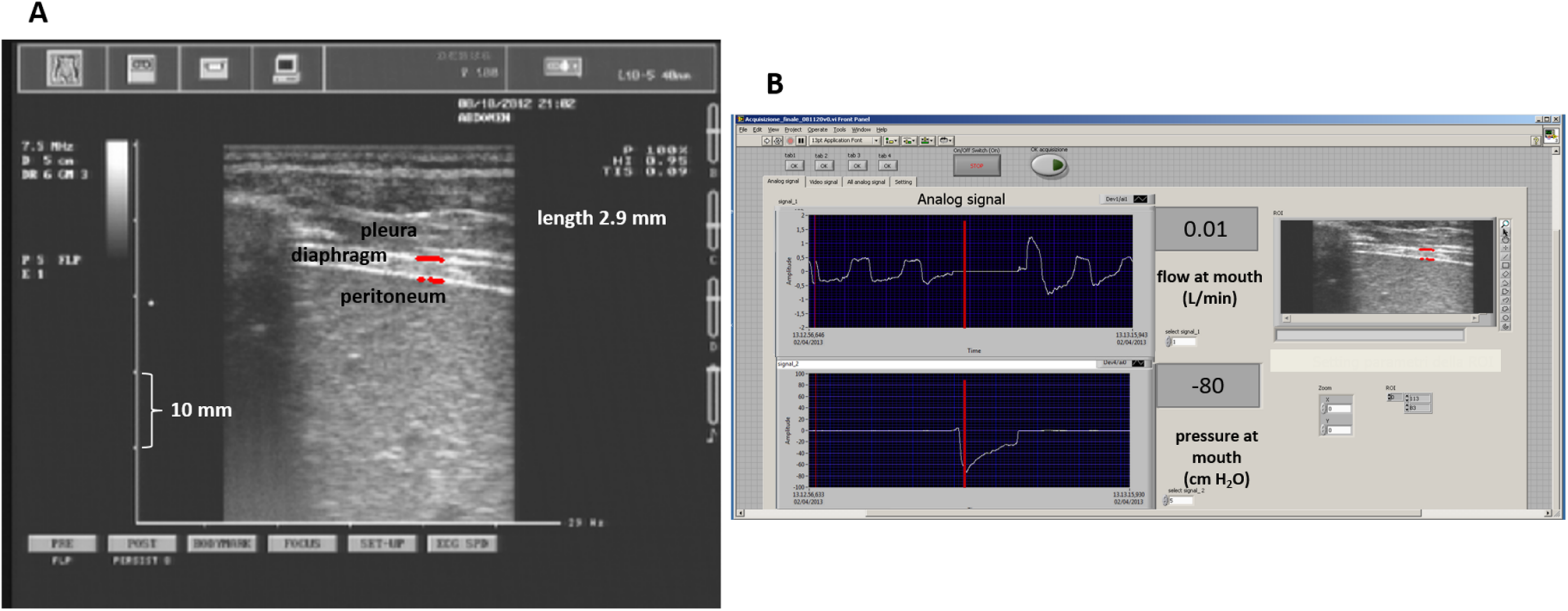
Diaphragm B-mode ultrasound and acquisition software front panel. **(A)** Diaphragm thickness (DT) at maximal inspiratory pressure maneuver in supine position measured as the distance between pleural and peritoneal membranes of the diaphragm colored in red; (B) front panel of the Labview software for the synchronously recording of ultrasonographic images and flow and pressure signals.

All ultrasonographic images, coming from the echograph, and flow and pressure analogic signals were recorded synchronously at a sampling rate of 10 and 200 Hz, respectively, by a custom-designed Labview^®^ software connected to an A/D board (National Instruments USB-6008 DAQ) (Fig 1). Images were saved into series of raw bitmap files.

### Image and data analysis

Processing of images and signals was performed offline by a custom software developed in Matlab^®^. The software allowed to select specific instants on pressure or flow tracings and to load the corresponding image, i.e. the saved image corresponding to the closest sample to the instant of interest. More specifically, we considered zero-flow points during quiet breathing (i.e., end expiration, EE, and end-inspiration, EI) and the instant at maximal inspiration during an inspiratory capacity maneuver, chosen as the maximal value on the time-integrated flow signal (here below called TLC). The points in which the pressure reached the minimum value during a maximal inspiratory pressure (MIP) maneuver and the maximum value during a maximal expiratory pressure (MEP) maneuver were also selected for analysis and called MIP_US,max_ and MEP_US,max_, respectively.

Once the time-instant of interest was selected, the software performed the following analysis:

1. automatic extraction of the two curves corresponding to the edge of the pleural and the bottom of the peritoneal membrane of the diaphragm, on the basis of a region growing algorithm and a set of morphological operators applied to the US image [18, 19];
2. calculation of DT, as the average distance between the two curves extracted on the US image;
3. saving of the values of volume variations (obtained by integration in time of flow signals) during QB and IC maneuvers, and of pressure, during MIP and MEP maneuvers.

The shortest distance that could be resolved between the two lines using this approach was 0.5 mm, a value significantly lower than the thickness of the relaxed diaphragm.

On each subject, DT was assessed by considering the average value of five breaths (EE and EI), and between 2 and 4 acceptable IC, MIP and MEP manoeuvers.

Three different measurements of maximal inspiratory pressure (MIP) and maximal expiratory pressure (MEP) expressed in cmH_2_O were recorded:

1. MIP_US_ and MEP_US_: pressures measured during MIP and MEP manoeuvers performed in supine position, and recorded during ultrasound measurements, occurring at the time when diaphragmatic thickness was obtained on US images;
2. MIP_US,max_ and MEP_US,max_: maximal pressures recorded during MIP and MEP manoeuvers, in supine position during ultrasound measurements;
3. MIP_seat_ and MEP_seat:_ maximal pressures measured during MIP and MEP manoeuvers in seated position, performed on the same day of ultrasound measurements.

For the calculation of the predicted values of MIP and MEP, the following equations, valid for boys, were used: MIP_pred_ = [2.58 + age x 0.39] [20] and MEP_pred_ = [35+ (5.5 x age)] [21].

### Statistical analysis

The sample size calculation was performed considering diaphragm thickness data obtained in a previous study [11]. We considered a significance level of 95% (p < .05), 80% power, a standard deviation of 0.15 mm and a minimum calculated detected difference of 0.2 mm. The sample size was estimated as 10 subjects according to these data.

To evaluate healthy controls and DMD patients’characteristics and DMD patients’ pulmonary function tests among tree groups (gathered by age), a one-way analysis of variance (ANOVA) was performed; while to compare within each group MIP_US_, MIP_USmax_ MIP_seat_ and MIP%pred a one way repeated measures was applied.

To determine the difference in DT between overall DMD patients and healthy controls a two-way ANOVA was performed, using disease (healthy controls and DMD) and maneuver (EE, EI, TLC, MEP and MIP) as independent factors. To evaluate the difference in DT among all three DMD groups and controls, a two-way ANOVA was performed, at EE, EI and MIP, using disease and age as independent factors.

Parametric tests were performed when variables were normally distributed, otherwise non-parametric tests were used. For multiple comparisons *post hoc* tests based on Holm-Sidak method were used. Sample sizes were calculated during quiet breathing and maneuvers in both healthy controls and DMD patients, by choosing a value of 0.05 and a desired power of 0.80. Differences were considered as significant with p value <0.05. Statistical analysis were performed using the software SigmaStat 3.5.

## Results

The anthropometric characteristics of DMD population are reported in Table 1.

**Table 1:** Duchenne muscular dystrophy (DMD) patients’ characteristics and pulmonary function test results.

	All	age<14yrs	age14-18yrs	age>18yrs	p-value
**Healthy Controls**					
n	13	5	3	5	
Age (yrs)	15.2±6.5	8.6±2.1	14.3±2.5°°°	22.4±1.1°°°,~~~	<0.001
Height (cm)	162.0±21	134.5±3.3	175.3±4.5°°°	176.1±5.1°°°	<0.001
Weight (Kg)	55.5±23	28.8±2.5	62.3±9.7°	72.7±16.6°°°	0.001
BMI (Kg/m^2^)	20.2±4.3	16.3.8±2.3	20.2±2.1	23.3±4.1°	0.028
**DMD Patients**					
n	44	13	15	16	
Age (yrs)	16.3±4.6	11.0±1.53	15.53±1.36°°°	21.50±1.97°°°,~~~	<0.001
Height (cm)	155.4±16	134.9±8.9	159.9±10.2 °°°	167.8±6.1 °°°,~~	<0.001
Weight (Kg)	54.2±18.6	40.2±12.4	62.0±17.8°°	58.5±17.7°°	0.004
BMI (Kg/m^2^)	22.2±5.1	21.7±5.2	23.9±4.6	20.7±5.3	NS
**Spirometry**					
n	42	11	15	16	
FVC (%pred)	53.4±24.6	78.3±16.9	55.2±22.9°°	34.5±11.8°°°,~~	<0.001
FEV1 (%pred)	51.8±28.5	84.4±22.3	49.1±22.9°°°	31.9±13.6°°°,~	<0.001
FEV1/FVC (%)	77.7±20.1	82.1±26.6	74.9±21.3	77.2±13.6	NS
PEF (%pred)	44.0±24.2	65.3±28.7	44.6±17.1°	28.8±13.9°°°,~	<0.001
**Respiratory muscles pressures**					
n	39	10	15	14	
MIP (cmH_2_O)	31.4±17.8	35.0±12.9	35.9±23.0	23.9±12.3	NS
MIP%pred	37.2±23.8	52.4±22.3	41.4±26.2	21.8±10.7°°°	0.001
MEP (cmH_2_O)	31.0±13.6	40.9±11.4	30.0±14.5	25.2±10.6°	0.023
MEP%pred	25.2±15.9	39.7±19.5	24.8±11.8	15.3±7.6°°	0.003
**Lung Volume**					
n	42	11	15	16	
TLC (%pred)	63.9±24.9	81.2±19.1	65.5±21.8	49.7±23.8°°	0.004
RV (%pred)	102.1±51.9	107.6±61.2	98.7±40.9	107.6±61.2	NS
FRC,N_2_ (%pred)	72.4±29.7	81.1±31.2	69.2±25.0	69.1±33.3	NS
**Sp,O**_**2**_%					
n	42	12	15	15	
100–95	91.7±15.8	99.7±0.9	89.9±19.2°	88.0 ±16.9°°	0.002
94–90	8.0±15.8	0.3±0.9	9.5±19.2°	12.5±17.0°°	0.002
<90	0.2±0.8	0.0±0.0	0.5±1.4	0.1±0.35	NS

(n: available data; NS: nonsignificant, Sp,O_2_%: percentage of the night-time spent with Sp,O_2_ in different ranges 95–100%, 90–94%, <90%). Data are presented as mean±SD.

p-values (one-way ANOVA): °,°°,°°°,: p<0.05, p<0.01, p<0.001 vs age<14; ~,~~~, ~~~: p<0.05, p<0.01, p<0.001 vs age 14÷18

### Pulmonary function

The results of pulmonary function tests of DMD patients are shown as average values as a function of the different age groups in Table 1. A strongly significant progressive reduction of predicted FVC, FEV_1_, PEF (p<0.001) and TLC (p = 0.004) with age was observed. Similarly in the predicted values of MIP (p = 0.001) and MEP (p = 0.003) a marked decrease was observed, while MEP slightly decreases (p = 0.023). Nocturnal time spent in desaturation significantly increased with age (p<0.002)

### Maximal pressures

Table 2 shows all values of MIP_US_, MEP_US_, MIP_US-max_, MEP_US-max_, MIP_seat_ and MEP_seat_.

**Table 2:** Values of pressure during MIP and expiratory MEP maneuvers.

Pressure (cmH_2_O)	Controls subjects			DMD patients		
	age<14yrs	age14-18yrs	age>18yrs	age<14yrs	age14-18yrs	age>18yrs
MIP_US_	33.6±17.6	67.1±50.3	75.9±22.2	27.4±19.1	42.0±18.0	22.9±12.1
MIP_US,max_	39.3±23.3	77.6±58.9	83.4±18.4	30.8±19.6	43.4±10.6	23.9±10.7
MIP_seat_	N.A	N.A	N.A	35.4±12.9	35.9±23.0^$^	23.9±12.3
MEP_US_	25.3±16.1	58.7±11.6	78.7±18.8	22.2±9.3	18.8±11.3	17.7±9
MEP_US,max_	30.7±19.0	63.2±8.7	85.9±18.4^##^	23.6±8.9	20.9±12.1^#^	20.7±8.9
MEP_seat_	N.A	N.A	N.A	40.9±11.4^$ $ $, ###^	30.0±14.5^##^	25.2±10.6^$, ###^

Data are expressed in cmH_2_O and presented as mean±SD. MIP_US_ and MEP_US_: pressure values correspondent to the instant in which the US image was chosen in order to calculate diaphragmatic thickness (supine position); MIP_US,max_ and MEP_US,max_: maximal pressure values measured during the maneuver in which the US images were recorded in order to calculate diaphragmatic thickness (supine position); MIP_seat_ and MEP_seat_: maximal pressures recorded by a commercial respiratory pressure meter (seated position). p-values (one-way RM ANOVA): $,$ $ $: p<0.05, p<0.001 vs MIP_US,max_ or MEP_US,max_; #, ##, ###: p<0.05, p<0.01, p<0.001, vs MEP_US_;

For the majority of MIP measurements no significant differences were found (just in the age group 14–18 yrs, the values in seated position were significantly lower than in supine), whereas for MEP measurements the values obtained in the seated position were significantly higher than those in supine.

### Diaphragmatic thickness

**Fig 2:**
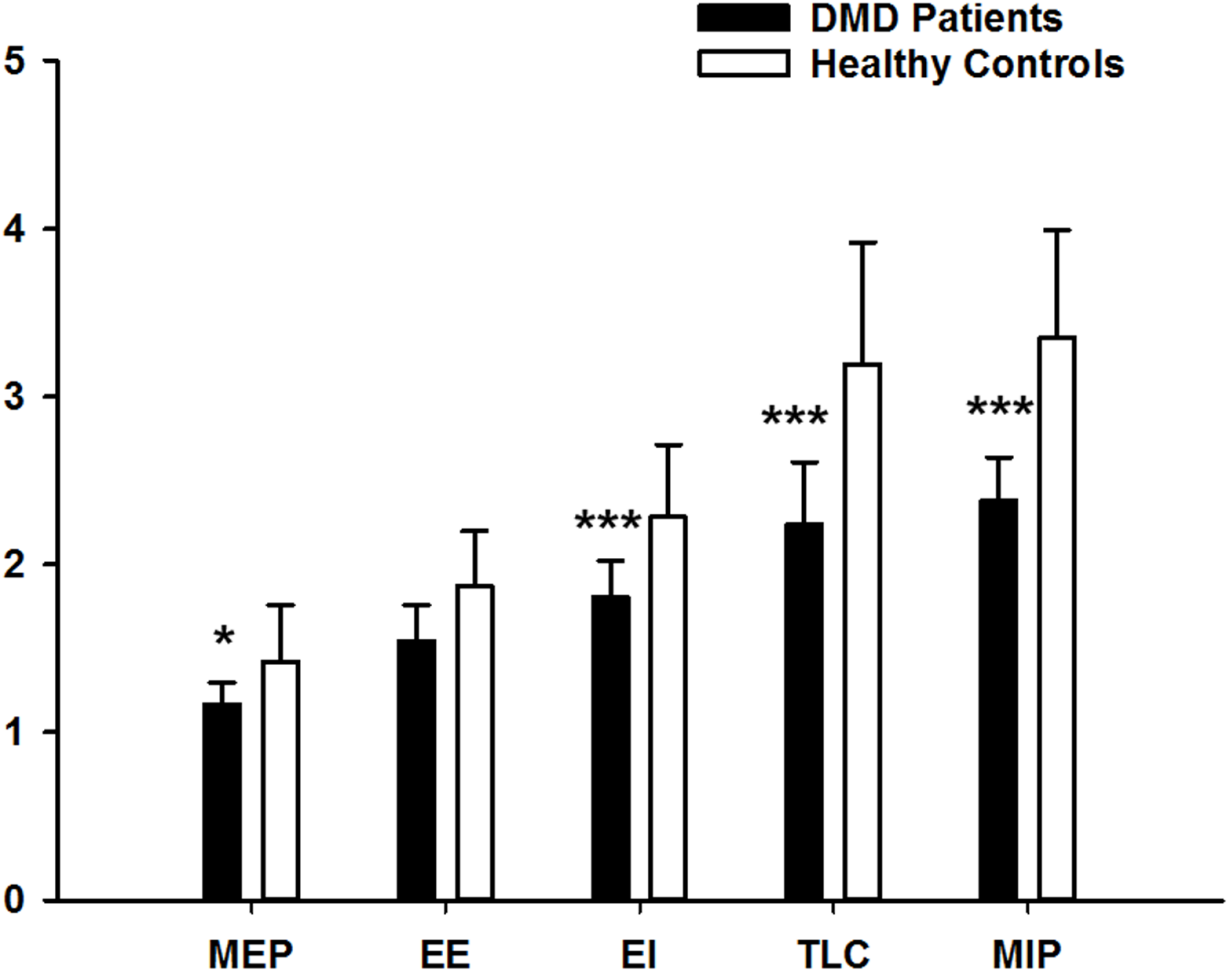
Average data of diaphragmatic thickness (DT) per overall groups. Values of DT are reported at end-expiration (EE) and end-inspiration (EI) during quiet breathing, at total lung capacity (TLC) during full inspirations and during maximal inspiratory (MIP) and expiratory (MEP) pressure manoeuvers in healthy controls (white bars) and DMD patients (black bars). (*,***: p<0.05, p<0.001 vs healthy controls).

As shown in Fig 2, the values of diaphragmatic thickness in all DMD patients were significantly lower than healthy controls in each considered conditions, apart from EE.

**Fig 3:**
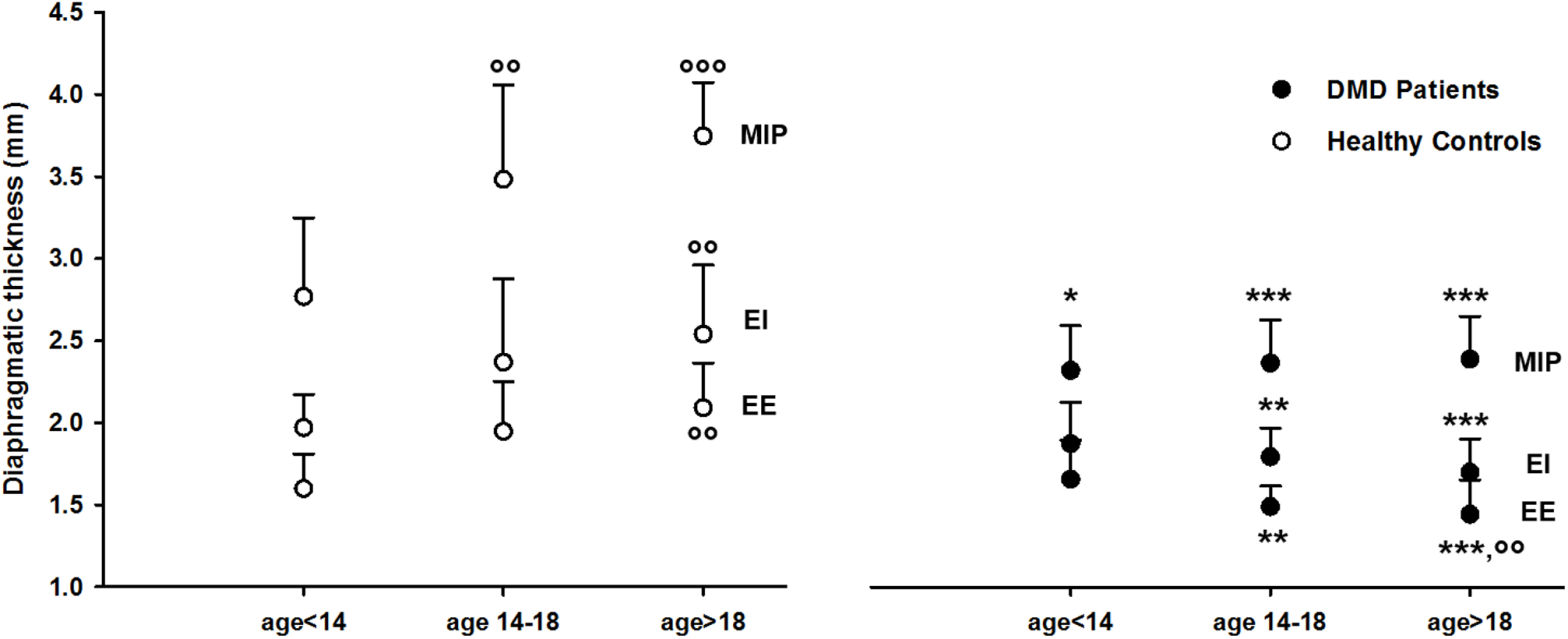
Diaphragmatic thickness (DT) per age group. Values of DT are reported at end-expiration (EE), end-inspiration (EI) and during maximal inspiratory pressure (MIP) maneuvers, in healthy controls (white circles) and in DMD patients (black circles) grouped by age (*,**,***: p<0.05, p<0.01, p<0.001 vs healthy controls, °°,°°°: p<0.01, p<0.001 vs age<14).

In Fig 3, all values of DT at EE, EI and MIP are shown for healthy controls (white circles) and for DMD patients (black circles) for the three age groups. In healthy controls diaphragmatic thickness significantly increased with age. Conversely, in DMD patients diaphragmatic thickness significantly decreased with age at end-expiration and it remained constant at end-inspiration and during MIP manoeuver. In the age groups 14–18 and >18 yrs, DT was significantly lower in DMD patients than healthy controls for all considered conditions. The complete set of average values of DT at EE, EI, TLC and during MIP and MEP for the different age groups of healthy controls and DMD patients is reported in Table 3. In the same table, also DT variations, expressed as percentage DT variations between TLC and EE and MIP and EE, are reported. Comparing healthy controls and DMD patients, all DT and DT variations were significantly different, with the only exception of DT values at EE and EI during quiet breathing and MEP in the age group <14 yrs.

**Table 3:** Complete set of average values of diaphragmatic thickness (DT) in healthy and in DMD patients.

Diaphragmatic Thickness (DT)	Control Subjects			DMD patients			
	age<14yrs	age14-18yrs	age>18yrs	age<14yrs	age14-18yrs	age>18yrs	p-value (control vs DMD)
DT at End-Expiration (EE), mm	1.6±0.2	1.9±0.3	2.1±0.3°°°	1.7±0.2	1.5±0.1**	1.5±0.2***,°°	<0.001
DT at End-Inspiration (EI), mm	2.0±0.2	2.4±0.5	2.5±0.4°°°	1.9±0.2	1.8±0.2***	1.7±0.2***	<0.001
DT at TLC, mm	2.8±0.4	3.3±1.2	3.5±0.6	2.1±0.6**	2.2±0.2***	2.3±0.3***	<0.001
DT at MIP, mm	2.8±0.5	3.5±0.6°°	3.9±0.3°°°	2.3±0.3*	2.4±0.3***	2.4±0.26***	<0.001
DT at MEP, mm	1.2±0.4	1.7±0.2°°°	1.4±0.3	1.2±0.1	1.2±0.1***	1.1±0.2**	0.004
ΔDT_TLC-EE_%	77.2±23.5	68.1±35.6	67.8±36.5	43.2±18.3**	48.6±19.4	43.2±15.5*	<0.001
ΔDT_MIP-EE_%	73.8±25.1	79.3±17.0	85.7±13.7	43.6±26.3*	59.4±24.5	56.3±13.4*	<0.001
TF%	21.3±12.1	20.8±8.5	23.9±9.4	18.4±10.1	20.4±9.5	15.5±4.9	NS
TR_TLC_	1.7±0.4	1.7±0.4	1.8±0.2	1.6±0.3*	1.5±0.2	1.2±0.5°°	0.023
TR_MIP_	1.9±0.1	1.8±0.2	1.7±0.3	1.4±0.6	1.6±0.2	1.4±0.3*	0.011

EE, end-expiration, EI, end-inspiration, TLC, full inspirations at total lung capacity, MIP maximal inspiratory pressure, MEP, maximal expiratory pressure; ΔDTTLC-EE% percentage variation of diaphragmatic thickness between TLC and EE: (diaphragm thickness at TLC- diaphragm thickness at EE) /diaphragm thickness at EE; ΔDTMIP-EE% percentage variation of diaphragmatic thickness between MIP and EE: (diaphragm thickness at MIP- diaphragm thickness at EE) /diaphragm thickness at EE; TF: thickness fraction: (diaphragm thickness at EI- diaphragm thickness at EE) /diaphragm thickness at EE; TRTLC, thickening ratio: diaphragm thickness at TLC/diaphragm thickness at EE; TRMIP, thickening ratio: diaphragm thickness at MIP/diaphragm thickness at EE; p-values (two-way ANOVA): *,**,***: p<0.05, p<0.01, p<0.001 vs control subjects; °°,°°°: p<0.01, p<0.001 vs age<14yrs; NS: non significant

### Relationship between DT and maximal pressures and DT and vital capacity

**Fig 4:**
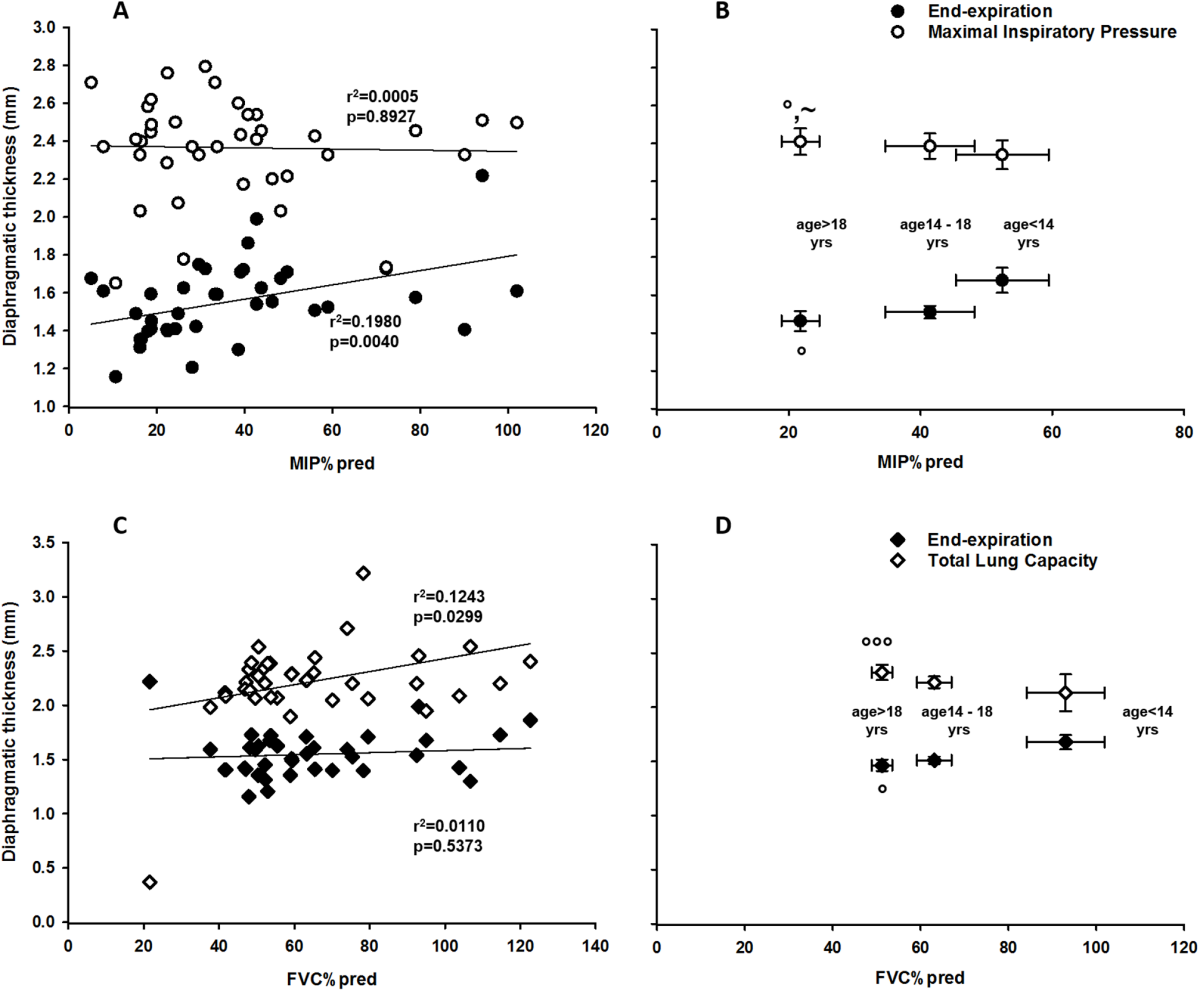
Diaphragmatic thickness (DT) related to MIP%pred and FVC%pred in DMD patients. DT at end-expiration (EE) (black circles), during maximal inspiratory pressure (MIP) maneuvers (white circles) and at total lung capacity (TLC) (white diamonds). A) Single values of DT for all DMD patients. A linear correlation was found only for DT in the EE condition. B) Mean values ± standard error of DT of the three DMD aged groups (°: p<0.05 vs group <14 yrs old; ~: p<0.05 vs 14÷18yrs); C) Single values of DT for all DMD patients. A linear correlation was found only for DT in the TLC. D) Mean values ± standard error of DT of the three DMD aged groups (**°°°** p<0.001 vs group <14 yrs old).

The existing relationship between diaphragmatic thickness and MIP%pred and diaphragmatic thickness and FVC%pred in DMD patients is reported in Fig 4 for both individual data (Fig 4A and 4C) and averaged over the different age groups (Fig 4B and 4D). Diaphragmatic thickness at EE was slightly correlated (r^2^ = 0.1980 and p = 0.0040) with MIP%pred whereas diaphragmatic thickness measured at MIP did not present any correlation. The age group >18 yrs was characterized by the lowest MIP%pred, the lowest averaged value of DT at EE and an average value of DT at MIP similar to those measured in the other age groups.

Diaphragmatic thickness at EE did not present any correlation with FVC%pred, whereas a slightly (r^2^ = 0.1243, p = 0.0299) correlation was found between diaphragmatic thickness at TLC and FVC%pred.

## Discussion

The first finding of the present study is that in our overall DMD population, diaphragmatic thickness is significantly lower than in controls in the majority of the analyzed conditions, namely at end-inspiration during quiet breathing, at total lung capacity and at maximal inspiratory and expiratory pressures.

A second finding is that, in the youngest DMD patients, diaphragmatic thickness at rest is similar to age-matched healthy controls with lower values of MIP, although not statistically significant. These results might suggest that the diaphragm is prone to pseudo-hypertrophy, however, future studies with a higher number of subjects are required to confirm this hypothesis, possibly using techniques with higher resolution than US. De Bruin et al. [11] described diaphragmatic pseudo-hypertrophy in young (10 years old) DMD patients, indicated by an increased diaphragmatic thickness at end-expiration. It can be hypothesized, therefore, that in the youngest DMD the diaphragm shows hypertrophy as other skeletal muscles and, similarly, it is associated to a reduction of the capacity of producing force. In our group of patients, in fact, we did not find a significant difference in DT between DMD and controls, however, the values of DT were lower in DMD compared to healthy controls considering the developed force (i.e., MIP).

A third relevant finding is that diaphragmatic thickness at end-expiration and end-inspiration was similar to the control group in the youngest (age<14yrs) DMD patients, but significantly lower in the middle (14-18yrs) and in the oldest (>18yrs) group of patients. This result is suggestive of a progressive atrophy of diaphragm muscle. Although no data are available regarding atrophy of the diaphragm in humans, a recent study performed on a canine (Golden Retriever) model of muscular dystrophy has shown evidence of morphometric remodeling of the diaphragm (i.e., loss of sarcomeres in series and increase in muscle stiffness) associated with rapidly progressive loss of ventilatory capacity after the first year of life [22].

The significance of DT measurements is different depending on the different considered parameters. Diaphragmatic thickness in the relaxed muscles (i.e. DT at end-expiration during spontaneous breathing) provides information regarding the total amount of muscle mass. Measurements of DT alone, however, may not discriminate between a paralyzed or functioning diaphragm. In fact, DT may be greater than 2.0 mm if the paralysis occurs and atrophy has not occurred. On the other hand, DT may be less than 2.0 in some individuals with a functioning diaphragm who have generalized muscle wasting or in small individuals [23].

Variation of DT during different manoeuvers (ΔDT), instead, provides information related to the number of active muscle fibers [23]. More specifically, diaphragmatic thickening during MIP manoeuver has formerly been shown to be a good indicator of the efficacy of inspiratory muscle contraction in producing force in normal adults [10]. In our population of both healthy controls and DMD patients, during MIP the variation of DT relative to rest (ΔDT%) and the thickness ratio were not significantly different in the different age groups, but lower than controls [11]. Furthermore, in healthy subjects MIP increases with age, while in DMD it decreases [24–26]. This result, moreover, suggests that diaphragm impairment in DMD could be expressed as a dissociation between muscle drive and muscle developed force, as recently shown by Burns et al. [27] in the mouse model of DMD, who found a potentiated neural motor drive to breathe suggesting compensatory neuroplasticity enhancing respiratory motor output to the diaphragm. This confirms that diaphragm weakness appears to develop, and to be more noticeable, at a later stage of the disease [28–32].

The present study has several strengths. Here, we considered a wide range of ages of DMD patients, including not only young patients, as in the previously performed studies, but also older ones, with age-matched healthy controls. Diaphragmatic thickness was evaluated during different maneuvers, such as quiet breathing, full inspiration, MIP and MEP.

Moreover, values of DT in healthy subjects at end-expiration (1.9 ± 0.3 mm) are in agreement with previous studies [10, 18] such as those during MIP maneuver in the oldest group (3.9 ± 0.3 mm) [10]. Boon et al. [33] reported values of DT at end-expiration higher with respect to those reported here (3.3±0.1mm *versus* 1.9±0.3 mm). This discrepancy could be explained by the fact that those authors studied subject with a mean BMI of 27.9 Kg/m^2^ whereas in our study the mean BMI was 20.2 Kg/m^2^. Nevertheless, the lower limit of DT (1.7 mm) and the thickening ratio during a full inspiration were consistent with the values found in the present study (1.8±0.5 vs 1.7±0.3).

Additionally, ultrasound images were synchronized with flow and pressure signals in order to calculate DT in the exact correspondence of the selected breath or maneuver. For this purpose, it was developed a dedicated system for simultaneous measurement of echographic images and respiratory signals (flow and pressure), which enabled a precise assessment of DT at the different relevant times. It is also important to note that MIP and MEP were measured not only in the experimental (i.e., supine during US measurements), but also in the clinical standard (i.e., seated) conditions.

Limitations of the study include the relative small number of healthy subjects. However, in our control group the values are very similar to those reported in the literature [34].

Also, DT measurements were performed only in supine position. This was due to difficulties in performing the measurements in other positions, such as the seated position in the wheelchair. In the seated position it is not-practicable to hold the probe perpendicular to the right chest wall. Nevertheless, supine position has been shown to be the best posture in order to show diaphragmatic impairment [30, 35]. In addition, as right hemidiaphragm is easier to observe than on the left due to its large contact with surface with the liver, we did not perform measurement at the left side of the diaphragm. Gottesman and Mc Cool [23], however, did not find any significant difference in DT measurements between the left and right hemidiaphragm. Lastly, we measured thickness in the costal region only. Thibaud et al. [36], however, showed by MRI a relative heterogeneity of diaphragm structural alteration in the Golden Retriever model of muscular dystrophy.

We believe that the study has a number of clinical implications. DT assessment by ultrasound has been already validated in healthy subjects [10, 37] and in supine position [9]. Moreover, it represents an inexpensive, noninvasive and easy method, available in all clinical centers, for assessing the progressive diaphragm involvement in DMD patients, to potentially be considered as outcome measure in clinical trials.
